# The Impact of ADHD on Children’s Language Development

**DOI:** 10.3390/children13020206

**Published:** 2026-01-31

**Authors:** Dimitra V. Katsarou, Asimina A. Angelidou

**Affiliations:** Department of Preschool Education Sciences and Educational Design, University of the Aegean, 85132 Rhodes, Greece; psed23009@rhodes.aegean.gr

**Keywords:** ADHD, language development, interventions, syntax, morphology

## Abstract

Background: This research explores the complex relationship between Attention Deficit Hyperactivity Disorder (ADHD) and language skills, focusing on the impact of the disorder on children’s language development. It is designed as a systematic literature review to synthesize and evaluate existing evidence on this topic. Based on the existing literature, ADHD affects multiple dimensions of language, including phonological awareness, pragmatic comprehension, morphosyntactic structure, narrative skills, and written expression. The difficulties that children with ADHD exhibit at the language level are directly related to their deficits in working memory, attention, and organization, which make it challenging for them to acquire and use language at both educational and social levels. Methods: This study followed the PRISMA methodology, with a systematic selection process across four stages (identification, screening, eligibility, and inclusion). During the identification phase, 475 records were identified (450 from database searches and 25 through reference screening). After screening and applying inclusion criteria, 15 studies met all eligibility requirements and were included in the final synthesis. Results: The present research highlighted the important role that occupational therapists and psychologists can play in the language development of children with ADHD. Strategic interventions to alleviate the language difficulties of children with ADHD are designed to enhance phonological awareness, executive function, speech and language, the use of technological tools, and social skills training. Conclusions: The importance of early diagnosis and implementation of holistic, individualized interventions targeting the language, executive, and social difficulties manifested by children with ADHD is considered influential in addressing the barriers to improving language skills as effectively as possible.

## 1. Introduction

Attention Deficit Hyperactivity Disorder (ADHD) is one of the most common neurodevelopmental disorders worldwide [1]. ADHD is characterized by symptoms such as difficulty sustaining attention, the onset of hyperactive behavior, and increased recklessness [2], usually manifesting before the age of 12 years. From an early age, children with ADHD are reported to have problems with language [3] and population-based studies suggest that the risk of having language problems is at least three times higher in children with ADHD compared to community controls [4]. Both the treatment and understanding of ADHD require a multidimensional approach, as the consequences of the disorder are wide-ranging and vary considerably from person to person [5].

In addition to the difficulties experienced by individuals with ADHD in terms of cognitive functioning and social behavior [6], the disorder has been shown to have a significant impact on language development, which is regarded as particularly important for children’s overall cognitive, educational, and social development critical for both academic and social success [7,8]. Language development challenges faced by individuals with ADHD include difficulties in understanding and using language, storytelling, and producing complex language structures [4,9].

Over the past decade, an expanding body of research has underscored the multifaceted relationship between Attention Deficit Hyperactivity Disorder (ADHD) and language development in children. Empirical findings indicate that children with ADHD frequently encounter difficulties across various linguistic domains, including phonology, morphology, syntax, semantics, and pragmatics, as well as in the dynamic interplay among these components [10]. Despite accumulating evidence, the precise mechanisms through which ADHD affects language acquisition and structure remain insufficiently delineated [11,12,13], thereby warranting a comprehensive synthesis of existing knowledge and the identification of emerging patterns.

From a theoretical perspective, two broad explanatory frameworks have been proposed to account for language difficulties in children with ADHD. Domain-general models suggest that language impairments emerge primarily as secondary consequences of deficits in attention, executive functioning, and working memory, which constrain language processing and use [9,11,14]. In contrast, domain-specific or hybrid accounts propose that at least some linguistic vulnerabilities may be partially independent of executive deficits, reflecting overlapping but distinct developmental pathways [15,16,17]. The present review adopts a critical, integrative perspective, using these competing models as an interpretive framework for synthesizing findings across language domains.

In light of this, the present article systematically reviews the extant literature on the impact of ADHD on children’s language development [18]. It examines the interrelations among core linguistic domains, including phonology, morphology, syntax, semantics, pragmatics, and general language abilities, identifies methodological and theoretical limitations in current research, and highlights the pivotal role of interdisciplinary professionals, including occupational therapists and psychologists, in supporting language development [15,19]. The review focuses on studies published from 2013 onwards, corresponding to the publication of the American Psychiatric Association (2013) [2], in order to capture research applying current diagnostic criteria and contemporary assessment tools. Furthermore, the review bridges theoretical insights with practical applications by considering factors such as ADHD subtype and comorbid conditions that may moderate outcomes and proposes evidence-based recommendations for interventions aimed at enhancing linguistic competencies. Finally, the review explicitly addresses the following aims: (1) to synthesize current evidence on language difficulties in children with ADHD; (2) to examine relationships between language deficits and cognitive, social, and academic outcomes; (3) to evaluate interventions targeting language development; and (4) to assess methodological quality and risk of bias, thereby guiding clinical practice and future research.

## 2. Methodology

The present study employed a systematic literature review approach, following the principles of the PRISMA framework, in order to critically examine the relationship between Attention Deficit Hyperactivity Disorder (ADHD) and language development in children. The methodology was designed to ensure transparency, replicability, and rigor in the identification, selection, and synthesis of relevant research.

### 2.1. Search Strategy

A comprehensive search of the academic literature was conducted across multiple electronic databases, including PubMed, PsycINFO, Scopus, Web of Science, and Google Scholar. These databases were selected as they provide extensive coverage of peer-reviewed publications in psychology, psychiatry, education, and allied health sciences. The search was guided by carefully constructed Boolean expressions to maximize sensitivity and specificity. Keywords included: “Attention Deficit Hyperactivity Disorder” OR “ADHD” AND (“children” OR “adolescents”) AND (“language development” OR “phonology” OR “morphology” OR “syntax” OR “semantics” OR “pragmatics” OR “executive functions” OR “interventions”).

The time frame of the search was restricted to studies published since the publication of DSM-5 (2013) and March 2025 to capture both seminal work and the most recent advancements. Notably, the publication of the DSM-5 in 2013 introduced updates such as extending the age of onset criterion from 7 to 12 years, which affects the identification and characterization of ADHD in children [2,20]. This timeframe ensures that included studies reflect contemporary methodological and clinical practices. Only articles written in English were considered. Additional records were identified by screening the reference lists of relevant systematic reviews and meta-analyses. A limited number of scoping reviews were also consulted and included only when they offered structured background information or identified research gaps not covered by primary studies. These reviews were used solely for contextual purposes and did not contribute empirical data to the synthesis.

#### Inclusion and Exclusion Criteria

Clear inclusion and exclusion criteria were defined prior to the review process to minimize bias. Studies were included if they examined children or adolescents (ages 3–18) formally diagnosed with ADHD according to standardized diagnostic criteria (DSM-IV, DSM-5, ICD-10/11). They also had to investigate at least one aspect of language development (phonology, morphology, syntax, semantics, pragmatics, narrative ability, or written expression). The review included 15 peer-reviewed studies examining language development in children with ADHD, comprising four systematic reviews or meta-analyses [11,21,22,23] and 11 empirical studies covering quantitative, qualitative, and mixed-methods designs.

Exclusion criteria were also clearly defined to further ensure the quality and relevance of the studies included in the review. Studies were excluded if they focused exclusively on adults with ADHD, if they did not have a confirmed ADHD diagnosis, if they were case reports, conference abstracts, opinion papers, or non-peer-reviewed sources. Additionally, articles not published in English were excluded from the review.

### 2.2. Study Selection

The selection process unfolded in four stages, following the PRISMA framework. During the identification phase, the initial database search yielded 450 records, with an additional 25 records identified through reference screening. After removing duplicates, 440 unique records remained and were screened by title and abstract for relevance. In the eligibility phase, 35 full-text articles were retrieved and carefully assessed against the predefined inclusion and exclusion criteria. Articles were excluded if they lacked a formal ADHD diagnosis, focused on non-linguistic outcomes, or demonstrated methodological weaknesses, which were defined as small or unrepresentative samples, absence of appropriate control groups, and use of non-validated assessment tools, insufficient statistical reporting, or unclear data collection procedures. Following these criteria, 15 studies met all eligibility requirements and were included in the final synthesis.

Data extraction was conducted using a structured template to ensure methodological consistency. For each study, information was recorded regarding bibliographic details (author, year, and country of study), sample characteristics (age, gender distribution, ADHD subtype, and comorbidities), methodological design (longitudinal, cross-sectional, experimental, quasi-experimental, or review), assessment tools used for ADHD diagnosis and language measurement, the specific language domains investigated (phonology, morphology, syntax, semantics, pragmatics, and written expression), as well as the main findings related to language difficulties, differential diagnosis, or intervention outcomes.

The analysis proceeded in two stages. First, the studies were categorized according to the five core domains of language development, with additional categories for executive functions, differential diagnosis, and therapeutic interventions. Second, a thematic synthesis was undertaken to identify patterns of evidence across studies, highlight methodological strengths and limitations, and pinpoint areas of consensus and disagreement within the literature. Particular attention was given to the role of occupational therapists and psychologists, recognizing their central contribution to intervention and support for children with ADHD.

### 2.3. PRISMA Flow

**Figure 1:**
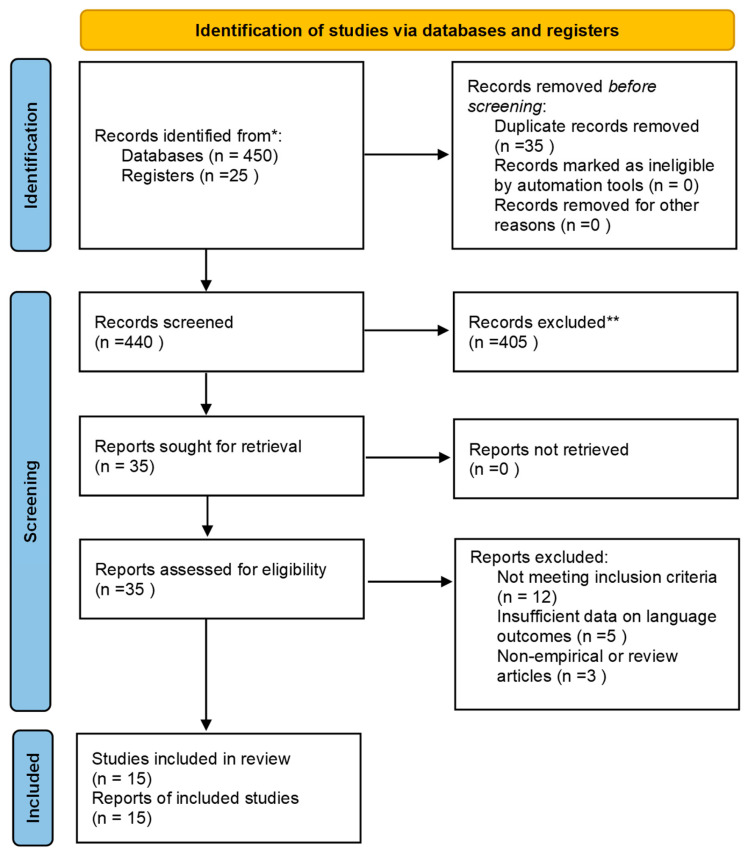
PRISMA flow diagram showing the study selection process. * indicates records identified through database searches; ** indicates additional records identified through manual reference-list searching.

The review process adhered to the PRISMA framework. The flow of information is summarized in the PRISMA diagram (Figure 1), which illustrates the number of studies identified, screened, assessed for eligibility, and finally included in the review. The diagram also records the number of studies excluded at each stage, together with justifications.

## 3. Results of the Systematic Review

### 3.1. Overview of Included Studies

A total of 15 studies were included in the final synthesis, covering a wide range of linguistic domains, including general language abilities, phonology, morphology, syntax, semantics, and pragmatics. The characteristics of these studies are summarized in Table 1, which includes details on study design, population characteristics, language measures, main outcomes, and risk of bias.

The included studies comprised observational (*n* = 10), meta-analytic (*n* = 2), systematic review (*n* = 1), and intervention studies (*n* = 2), all involving children with a formal diagnosis of ADHD. Participants ranged from preschool to school-aged children, with several studies targeting specific linguistic subdomains such as syntax comprehension, narrative skills, or pragmatic language.

The largest number of studies focused on general language difficulties (*n* = 3) and phonological processing (*n* = 3). Syntax (*n* = 2), pragmatics (*n* = 3), morphology (*n* = 1), and semantics (*n* = 1) were also represented. Across studies, children with ADHD consistently exhibited deficits in multiple language domains. Phonological and pragmatic difficulties were among the most robustly reported challenges, affecting both academic and social functioning. Intervention studies demonstrated that targeted programs could improve phonological processing, syntax comprehension, and overall language performance, supporting the potential for remediation in this population.

### 3.2. Risk of Bias and Quality Assessment

The quality of the included studies was evaluated based on methodological risk of bias, summarized in the Risk of Bias column in Table 1. Observational studies were generally rated as moderate risk of bias due to limitations such as small sample sizes, lack of control groups, or incomplete reporting of outcome measures. Meta-analyses and systematic reviews were rated low risk of bias because of transparent methodology and comprehensive literature synthesis. Intervention studies also received moderate ratings when randomization or blinding was limited.

Overall, 9 studies (60%) were rated as moderate risk of bias, and 6 studies (40%) were rated as low risk of bias, with no study in our current sample reaching high risk. The risk of bias assessment supports the reliability of the main findings, though variability in study design and methodological quality should be considered when interpreting the results.

It should be noted that no pre-registered review protocol was established prior to study selection, and a single standardized quality appraisal tool was not applied. Instead, risk-of-bias judgments were informed by transparent reporting, study design characteristics (e.g., sample size, presence of control groups, and clarity of outcome measures), and consistency of findings across studies. This approach aligns with prior narrative and mixed-methods systematic reviews in heterogeneous research fields, while acknowledging its inherent limitations.

### 3.3. Summary of Findings by Language Domain

General Language Abilities: Three studies [10,15,24] highlighted that children with ADHD exhibit broad deficits in expressive and receptive language, often correlated with attention and executive functioning difficulties.

Phonology: Three studies [25,26,27] reported phonological deficits, which were linked to early ADHD symptoms and reading difficulties. Intervention studies targeting phonological processing demonstrated measurable improvements.

Morphology: One meta-analytic study [11] confirmed consistent morphological deficits in children with ADHD across studies.

Syntax: Two studies [21,28] revealed difficulties in syntax comprehension, particularly in children with comorbid developmental language disorders.

Semantics: One study [29] reported impairments in pragmatic and semantic language skills.

Pragmatics: Two studies [22,30] emphasized significant pragmatic language challenges, affecting social communication and narrative abilities.

In conclusion, the synthesis of these 15 studies demonstrates that children with ADHD experience multifaceted language difficulties, with phonology and pragmatics being the most consistently affected domains. The combination of observational, meta-analytic, and intervention evidence suggests that these deficits are robust across populations and can be partially mitigated with targeted interventions. Notably, the strength and consistency of evidence varied across linguistic domains, with phonological and pragmatic impairments supported by more convergent findings than morphological and semantic difficulties [31].

Following the presentation of the overall findings in Figure 1 and Table 1, the subsequent section focuses on the domain of language development, which constitutes a fundamental aspect of neurocognitive functioning [24]. Given that language is intricately linked to attention, executive control, and working memory processes, examining this domain provides critical insights into the ways in which ADHD may disrupt the acquisition, organization, and use of linguistic abilities [32,33,34,35,36,37,38,39].

**Table 1:** Summary of Included Studies on Language Development in Children with ADHD.

Category	Article	Year	Study Type	Language Measures	General Main Outcomes	Risk of Bias
General Language Difficulties in Children with ADHD	[10]	2016	Observational	General language assessment	General language abilities assessed through standardized language assessments. Measured via FTF (Five to Fifteen) parent-report questionnaire.	Moderate
Phonology	[25]	2013	Observational	Early language/phonology tests	Assessed early phonological and language skills using observational early language/phonology tests.	Moderate
Morphology	[11]	2017	Meta-analysis	Various language tests	Language problems associated with ADHD. Using multiple standardized language tests (e.g., CELF variants).	Low
Syntax	[21]	2024	Systematic review	Syntax comprehension	Review of syntax skills in ADHD and DLD. Various standardized and domain-specific assessments reported across included studies (e.g., CELF-4, CCC-2, TOPL-2, NAP, TEGI, non-word repetition).	Low
Semantics	[29]	2016	Observational	Pragmatic/semantic tests	Assessed pragmatic and semantic language skills using observational pragmatic/semantic tests.	Moderate
Pragmatics	[22]	2023	Meta-analysis	Pragmatic/social language measures	Reported social and pragmatic language deficits using standardized and domain-specific pragmatic/social language measures across included studies.	Low
Pragmatics	[30]	2023	Observational	Structural & pragmatic language tests	Impact of ADHD on language skills. Assessed structural (vocabulary, grammar) and pragmatic language skills using LOGOMETRO in children with ADHD.	Moderate
ADHD and Language Development	[15]	2017	Observational	Language & social cognitive tests	Assessed language and social-cognitive skills using observational language and social cognitive tests.	Moderate
ADHD and Language Development	[12]	2022	Observational	Narrative language tests	Evaluated narrative language abilities using standardized narrative language assessments.	Moderate
ADHD and Language Development	[24]	2024	Observational	Language development assessments	Assessed overall language development using domain-specific language development tests	Moderate
Differential Diagnosis Challenges	[27]	2022	Observational	Phonological & reading tests	Assessed phonological and reading skills using standardized phonological and reading tests.	Moderate
Diagnostic Challenges in ADHD	[26]	2020	Intervention	Phonological processing tests	Evaluated phonological processing and intervention outcomes using phonological processing tests.	Moderate
Occupational Therapist Role	[28]	2025	Observational	Syntax comprehension	Assessed syntax comprehension and improvement via observational syntax tests in occupational therapy context.	Moderate
Psychologist Role	[40]	2013	Observational	General language assessment	Evaluated overall language skills using general language assessment measures.	Moderate
Interventions to Improve Language Development	[23]	2024	Observational/Intervention	Language & social/academic measures	Assessed language skills and their relation to social and academic functioning using combined language, social, and academic measures.	Moderate

## 4. Language Abilities in ADHD

The Language Development section provides a synthesized overview of the studies, highlighting key insights rather than summarizing each study individually. Language skills allow individuals to use their language accurately by conveying meaning, a condition that contributes to its production and comprehension [22]. According to [21], this ability is essential for personal and academic development. Research also shows that in children with ADHD, language skills are often impaired [11,23], affecting comprehension and communication in everyday contexts. The acquisition of language skills is critical for human communication and development, as it affects the individual on a personal, educational and professional level. Language skills are multidimensional since they are related to an individual’s ability to listen, speak, read, write, understand and use language in specific social and cultural contexts [31].

Phonology, morphology, syntax, semantics, and pragmatics are the five key linguistic domains that are considered necessary for an individual to understand and use language in an effective way. The five domains work together for the individual to form a dynamic and unified linguistic whole in order to communicate and understand, both at the level of spoken and written language, the people with whom he/she interacts [33].

### 4.1. Phonology

More specifically, regarding the five basic linguistic structures, phonology focuses on the abstract systems and phonological rules through which individuals shape the pronunciation of words. It forms the basis for morphology and syntax, as phonological constraints cannot affect the form and order of words [34]. According to [11], in children with ADHD, phonological processing is often compromised, particularly in tasks requiring fine-grained manipulation of sounds, such as phoneme deletion, blending, and nonword repetition. These deficits are not typically due to articulation problems but rather to limitations in phonological working memory and processing speed. For example, children with ADHD may struggle to hold multiple phonemes in mind simultaneously, resulting in slower or less accurate decoding of unfamiliar words.

Research by [27] indicates that phonological weaknesses in ADHD are especially pronounced when co-occurring with reading disorders, suggesting an additive effect of attentional and language deficits. Moreover, neuroimaging studies [35] have shown that children with ADHD display reduced activation in left-hemisphere regions associated with phonological processing, such as the superior temporal gyrus and inferior frontal gyrus, especially under tasks that require sustained attention. This indicates that phonological deficits in ADHD are closely tied to domain-general cognitive resources rather than isolated linguistic systems.

Recent work by by [30] highlights that interventions integrating phonological skill training with executive function supports—such as working memory scaffolding, attentional cues, and adaptive task pacing—lead to measurable improvements in decoding, reading fluency, and rapid naming speed. These findings suggest that phonological interventions for children with ADHD are most effective when they address both attentional and memory demands, rather than focusing solely on isolated sound discrimination exercises [30].

### 4.2. Morphology

Morphology deals with the structure of words and the rules of their formation, as it is shaped by phonological and syntactic constraints, while shapes can change the meaning and function of words through derivations and inflectional processes. Morphology affects semantics, as the morphological elements of a word cannot affect its meaning [36]. According to [10], ADHD-related morphological challenges are especially evident in contexts where words must be manipulated or integrated into complex syntactic structures. For example, producing past-tense forms in irregular verbs or deriving nouns from adjectives can impose additional cognitive load, revealing deficits in real-time morphological processing.

Research by [23] suggests that early weaknesses in morphological awareness in children with ADHD can contribute to later difficulties in reading comprehension and written expression, particularly when executive function skills are underdeveloped [12]. Furthermore, ref. [15] found that morphological difficulties in ADHD often coexist with broader language processing deficits, impacting both academic and social communication [15].

Interventions targeting morphology in children with ADHD are most effective when individualized and EF-sensitive. As highlighted by [24], techniques that scaffold attention, provide repetitive practice in meaningful contexts, and break down complex morphological rules into smaller steps can significantly improve both accuracy and retention [24]. Integrating morphological exercises with narrative or reading activities further enhances generalization, supporting both language comprehension and written expression [37].

Despite evidence suggesting morphological vulnerabilities in children with ADHD, the available literature remains limited in both scope and methodological rigor. Most findings derive from observational studies or broader language assessments rather than targeted morphological probes, restricting firm conclusions regarding the specificity and developmental trajectory of morphological difficulties in this population.

### 4.3. Syntax

Syntax focuses on the structure of sentences and the rules that determine how words are combined to form phrases and sentences. This system connects morphology and semantics, since it determines the relationship between words and their interpretation within a sentence. Syntax has been found to be related to morphology and semantics, as grammatical structure directly affects the semantic interpretation of a sentence. In addition, it can be influenced by morphological processes, such as rhythm and intonation, which affect the structure of sentences.

According to [28], in children with ADHD, syntactic processing can be particularly challenging when sentences involve complex or embedded structures, such as relative clauses, passives, or center-embedded constructions [28]. Research indicates that these difficulties often arise not from a primary syntactic deficit but from the interaction between syntactic complexity and domain-general executive functions, such as working memory, inhibitory control, and attentional allocation [38]. Jepsen et al. (2022) further support this, showing that ADHD children may correctly apply syntactic rules in simpler contexts but struggle when maintaining multiple elements in memory or when suppressing competing interpretations is required [23].

Cross-linguistic evidence, as reported by [28], illustrates that syntactic vulnerabilities in ADHD are modulated by language-specific properties. For instance, studies in Persian and other morphologically rich languages show that ADHD children exhibit greater difficulties in processing syntactic agreement and hierarchical embedding compared to typically developing peers, highlighting the cognitive load imposed by complex morphosyntactic integration [28]. Similarly, Chen et al. (2022) found that sentence comprehension is frequently impaired when tasks demand integration of multiple clauses or rapid parsing of ambiguous structures, demonstrating that syntax in ADHD is highly sensitive to attentional and processing constraints [27].

### 4.4. Semantics

Semantics refers to the study of the meaning of words, phrases, and sentences, focusing on the way concepts are organized at the linguistic level and how their meaning is conveyed through grammatical and lexical elements. It has been found that semantics interacts closely with morphology and syntax, since semantic categories determine how morphemes and syntactic structures are used to convey specific meanings [39]. According to Cohen et al. (2017), children with ADHD often experience challenges in semantic organization that influence how they process and interpret linguistic meaning in context [15].

In children with ADHD, semantic processing can be affected, particularly in tasks requiring rapid lexical access, integration of meaning across sentences, or inference-making. Jepsen et al. (2022) observed that these difficulties often emerge not from a lack of knowledge of word meanings but from deficits in attention, working memory, or executive control, which can slow semantic retrieval or reduce the efficiency of integrating information across sentences or discourse [23]. Vassiliu et al. (2023) further highlight that this leads to incomplete understanding, vague or imprecise expression, and reduced narrative coherence in everyday communication [30].

Interventions that support semantic development in ADHD often combine rich lexical instruction with scaffolds for executive function. Studies by [40] show that strategies such as explicit teaching of word meanings, semantic mapping, repeated exposure in context, and the use of visual organizers can improve both vocabulary depth and comprehension [24]. Additionally, Docking, Munro, & Cordier (2013) emphasize that integrating semantic-focused activities with attentional supports—like chunking or guided practice—further enhances learning outcomes, especially in children who show overlapping language and attentional weaknesses [40].

Importantly, semantic difficulties in ADHD are less consistently documented than phonological or pragmatic impairments and are often inferred indirectly through narrative or discourse-level tasks. This limits the precision with which semantic deficits can be distinguished from broader attentional or executive constraints.

### 4.5. Pragmatics

Finally, pragmatics focuses on the way language is used in different communicative contexts and specifically on how speakers adapt their linguistic production, depending on the context, social conditions, and relationships between interlocutors. It has been found that pragmatics interact with syntax and semantics, as linguistic expressions acquire different meanings depending on their environment [41].

Children who have well-developed language skills tend to develop better interper-sonal relationships, as well as a greater ability to express their thoughts and feelings. Together, the acquisition of language skills has been linked to levels of self-esteem and in-dependence. Also, children with well-developed language skills tend to perform better in school and cope with learning challenges with greater ease [42,43]. At the same time, children who have developed language skills are facilitated in terms of social inclusion, resulting in a higher level of psychological well-being and a reduced risk of developing anxiety [44].

It has been found that, particularly in the early developmental stages, language de-velopment has a significant impact on an individual’s cognitive and social progress. The challenges that children may face in acquiring language skills due to neurodevelopmental disorders have been found to affect both their academic performance and their social in-teractions [45,46].

## 5. ADHD and Language Development

ADHD often affects children’s language development, as it has been found that children with ADHD manifest various difficulties in understanding and using language, from delayed vocabulary development to problems in producing coherent and organized speech [14,47]. Specifically, as shown by Goldstein & Naglieri (2014), these challenges reflect the multidimensional nature of language skills, including phonology, morphology, syntax, semantics, and pragmatics [31].

Although the language difficulties experienced by children with ADHD are independent of their cognitive abilities [15], they nevertheless often face barriers related to expression, comprehension, language pragmatics [17], and morphosyntactic difficulties, resulting in many barriers to language acquisition [17,30]. Research by Long (2024) further indicates that co-occurring reading disorders intensify these deficits, pointing to an additive effect of attentional and language challenges [48].

More specifically, research has shown that the pragmatic deficits exhibited by children with ADHD are often interconnected with the language challenges they face, which in turn are related to both social interactions and cognitive functioning [49,50]. As highlighted by Parks et al. (2023), early pragmatic difficulties can have long-term impacts on social development and psychological well-being [45]. At the same time, the barriers that these children present in terms of executive functions do not allow them to interact effectively in various forms of language behavior, such as, for example, dialogue and storytelling [51].

Research suggests that 35% of children with ADHD have significant difficulties in maintaining visual contact and understanding social cues, resulting in a negative impact on their pragmatic and language skills [25]. According to [22], these difficulties are compounded by deficits in attentional control and phonological processing, limiting the integration of language components. At the same time, research has shown that children with ADHD have significant deficits in morphosyntactic as well as pragmatic levels [28,38,52].

According to Redmond & Ash (2014), children with ADHD have a high level of language difficulties compared to their peers without the disorder [16]. These difficulties include delays in vocabulary development, comprehension, and language use, problems with storytelling, and social communication. The barriers impede the child’s ability to develop coherent and organized speech, resulting in significantly affected academic performance [10], and they often experience social rejection [4].

It is estimated that approximately 30% of children with ADHD have significant difficulties in reading [27,53], and 40% in phonological processing [12,54]. The difficulties are particularly pronounced in the case of children who fall into the combined ADHD-Y type [55]. These challenges are further compounded since children with ADHD-Y are characterized by a poor vocabulary, have difficulty learning new words, use simple sentence structures, omit sentence elements, avoid complex expressions, and are characterized by a slow speech rate. The persistent nature of the aforementioned difficulties is exacerbated by the underlying symptoms that children with ADHD exhibit, in particular attention span and executive function deficits [54,56,57]. Attentional distraction prevents children with ADHD from mastering language skills as they are unable to effectively observe and imitate speech patterns from the social context in which they operate [54]. Furthermore, children with ADHD have difficulty pronouncing and recognizing phonemes accurately, resulting in barriers at the level of word pronunciation and issues related to lexical rhythm, a condition associated with sound processing problems and working memory deficits [57].

Children with ADHD also face many challenges in terms of written language, as they cannot easily express their knowledge, thoughts, perceptions and feelings. 45% of children with ADHD manifest significant difficulties in written expression, with texts characterized by brevity and limited coherence [57,58,59]. These difficulties are further exacerbated by attention deficits, as children with ADHD find it difficult to follow complex instructions and complete academic tasks, resulting in a limitation of their academic productivity and, by extension, their school performance [60].

### 5.1. The Difficulty of Applying Differential Diagnosis in the Assessment of ADHD

The differential diagnosis in the case of ADHD requires a multidimensional assessment, which includes the historical, psychological and educational history of the assessed, as well as clinical interviews [16,60,61]. However, the differential diagnosis is faced with a multitude of challenges, such as comorbidity and overlapping symptoms [15,62]. Specifically, children with ADHD show a high degree of comorbidity with other psychiatric disorders, which makes it difficult to distinguish between them [49,61,62]. It has been found that 70% of children and 50% of adults with ADHD simultaneously suffer from one of the following disorders, such as anxiety disorders, mood disorders, oppositional defiant disorder, autism spectrum disorder, learning disabilities, sleep disorders, or depression [62].

Also, one of the biggest obstacles to diagnosing ADHD is that many of its characteristics overlap with other disorders. For example, the impulsivity and hyperactivity of ADHD are often confused with mania in the case of bipolar disorder [61]. Attention deficit hyperactivity disorder can also occur in depression or anxiety disorders [62]. At the same time, children with ADHD have difficulty regulating their emotions, and as individuals they also experience mood disorders [63]. Accordingly, social interaction problems have been found to occur in both children with ADHD and children with Autism Spectrum Disorder [64,65].

In addition to the above, the differential diagnosis is faced with a lack of objective biological markers, as there are no hematological or neuroimaging tests that can diagnose the disorder with certainty [19]. At the same time, age differences are observed as the symptoms of the disorder manifest differently in children and adults with ADHD [62]. For example, adults show more inattention than hyperactivity. At the same time, the differential diagnosis can be influenced by cultural factors, such as the way in which the disorder manifests itself depending on the cultural context [62].

In order to overcome the aforementioned obstacles, the scientific community promotes the use of multiple diagnostic tools, such as structured interviews, assessment questionnaires and analysis of behavior in different settings, in order to minimize the risk of any misdiagnosis [16,66,67,68]. At the same time, newer research suggests differentiated diagnostic protocols that consider the neurobiological and psychological parameters of each individual [22,50,62]. For example, the use of highly accurate tools such as the Conners Rating Scale and Vanderbilt ADHD Diagnostic Rating Scale is recommended [66]. At the same time, it is recommended to conduct a neuropsychological assessment to identify the patterns of executive function that differ between ADHD, anxiety and mood disorders [67]. Finally, the scientific community suggests observing the assessed in different settings (home, school, work) [68].

It is understood from the above that the differential diagnosis of ADHD is a complex process due to the morbidity and overlap of symptoms with other disorders. Specialists must apply multiple diagnostic approaches to ensure an accurate and reliable diagnosis [62,69].

### 5.2. Diagnostic Challenges in ADHD Due to Comorbidity and Symptom Overlap

Diagnosing Attention Deficit Hyperactivity Disorder (ADHD) is often complicated by the high rate of comorbid conditions. Recent studies indicate that up to 80% of adults with ADHD present with one or more additional disorders, including anxiety, depression, substance use disorders, and oppositional defiant disorder [69,70]. This overlap of symptoms—such as inattention, hyperactivity, and impulsivity—makes it difficult to distinguish ADHD from other conditions, particularly when the inattentive presentation mimics anxiety or depressive disorders [71,72]. The co-occurrence of multiple disorders can also exacerbate functional impairments, leading to more pronounced academic, social, and behavioral difficulties [73,74]. Additionally, evolving diagnostic criteria, such as those in the DSM-5, allow for dual diagnoses, further complicating differentiation between primary and secondary symptomatology [75,76]. Accurate identification requires comprehensive assessment strategies, including multi-informant behavioral observations, standardized rating scales, and consideration of developmental history [51,68,74]. In sum, the substantial symptom overlaps and frequent comorbidities necessitate a careful, nuanced approach to ADHD diagnosis to ensure that treatment plans address all relevant conditions and minimize misdiagnosis or under-identification [71,72,75].

## 6. Interventions to Improve Language Development

The evidence base for interventions targeting language development in children with ADHD is methodologically heterogeneous. While some findings derive from structured intervention studies, including targeted phonological, executive-function, and language-based programs, a substantial proportion of the literature is based on observational, descriptive, or practice-informed research. Consequently, the effectiveness of interventions should be interpreted with caution, taking into account differences in study design, sample characteristics, and outcome measurement. Considering the diverse effects of ADHD on children’s language development, targeted interventions are needed to improve the level of language skills while addressing the specific needs of each child [5].

More specifically, it is necessary to implement interventions that focus on enhancing the phonological awareness of children with ADHD, due to their difficulties in recognizing letters and sounds, which directly affects the level of their reading skills [26,30,77]. Targeted intervention at the level of phonological processing leads to significant improvements in children’s reading ability [78]. At the same time, engaging in programs that incorporate tasks that address vowel and consonant recognition and management, as well as understanding syllabic word structure, has demonstrated highly positive results in the language improvement of children with ADHD [26]. The exercises help children to better understand how words are composed of sounds, thereby improving both their reading ability and language fluency. At the same time, educational approaches that focus on direct teaching of cognitive strategies, such as phonetic decoding and the use of mnemonic sound rules that contribute to memorization, have been found to help the developmental progress of children with ADHD [78].

Interventions that focus on the executive functions of children with ADHD are also considered important because of the deficits in working memory, attention and organisation that have a negative impact on language development and academic performance [79]. It has been found that children who participate in programs that focus on addressing challenges through engagement in targeted activities significantly improve their academic performance [80].

Also, engaging children with ADHD in activities that improve working memory and attention skills, such as memory games and multitasking exercises, positively contributes to managing language demands [31]. Teaching children with ADHD to organize using visual reminders and journals has been found to be instrumental in helping them to plan and complete tasks more efficiently [31,81]. Concomitantly, incorporating interventions into the school environment, such as providing regular feedback and teaching learning strategies that involve executive functions, has been found to contribute to self-regulation and academic performance of children with ADHD [81].

Also, interventions that focus on speech and language therapy are considered crucial factors to improve children’s oral and written expression as well as language comprehension [82]. Programs that focus on enhancing storytelling and organizing ideas can help children with ADHD develop coherent and language structures [55,83].

The process helps to improve the ability of the individuals involved to structure and express their thoughts clearly, enhance language fluency and narrative ability, elements that are particularly important for academic success and social interaction [77]. At the same time, the incorporation of individualized programs that provide children with ADHD the opportunity to practice using language in real-life settings has been found to be particularly effective in improving the programmes include word reading, spelling exercises and practices that develop children’s ability to follow directions and answer questions [84].

Often the use of technology is an important intervention to improve the written expression of children with ADHD, as it has been found that technological advances can address a variety of challenges related to spelling, grammar and language structure issues. Word processing programs with spelling and grammar checking capabilities help children with ADHD to identify and correct their errors while enhancing the quality of written work [85,86,87].

Similarly, voice recognition software programs allow children with ADHD to dictate their thoughts orally and convert them into written language. This technique is considered particularly beneficial for children who have difficulties with fine motor skills or difficulties with writing speed [85,86,87]. The use of tools such as the above-mentioned ones reduces the fatigue and frustration that can occur during written production, allowing children with ADHD to focus on the content and structure of their text [86,87]. Parallel software programs such as graphic organizers and text design tools, contribute to the organization of ideas and achieving better coherence of writing [88]. Research has shown that integrating technology tools into the school context contributes to significant improvements in the written expression and self-confidence of children with ADHD. Also, dividing written tasks into smaller, more manageable chunks can also help maintain the concentration and productivity of children with ADHD, resulting in significant improvements in the quality and coherence of written language [57].

Social skills training is a particularly effective intervention for children with ADHD, which, among other things, helps them effectively deal with difficulties in understanding informal communication rules, such as taking turns in speaking, maintaining eye contact, and understanding the emotions and intentions of other people [49,82,89]. Special educational programs related to social skills focus on strengthening pragmatic language, which includes the use of language in various social contexts, as well as the understanding of verbal communication elements, such as facial expressions and tone of voice [89]. At the same time, by incorporating exercises that enhance the understanding of social signals, the level of linguistic exchange is improved, allowing children with ADHD to participate in conversations with greater comfort and clarity. Improving social skills helps to cultivate verbal comprehension and overall communication in children with ADHD [82].

### 6.1. The Role of the Occupational Therapist in Improving the Language Development of Children with ADHD

Considering that ADHD is a neurodevelopmental disorder, apart from language development, it affects the individual in many ways in terms of communication skills, language structures and understanding of social skills [24,49,90,91]. It is therefore clear that the occupational therapist plays a crucial role, as through his or her specialised interventions, he or she enhances not only language skills but also the communicative and social interaction of these children [90].

By utilizing techniques that promote language and development through activities, occupational therapists work to enhance executive function, concentration and social interaction [91]. More specifically, by engaging children with ADHD in playful interventions, occupational therapists teach them to express themselves in the correct way, enrich their vocabulary and understand a plethora of social and communicative cues [40]. According to research results, it had been found that engaging children with ADHD in role-playing games and board games with rules improves their level of concentration and communication skills [92]. Also, by designing activities that involve interaction with peers, occupational therapists help these children improve their language skills through social interaction [40].

At the same time, occupational therapy has emerged as a particularly useful approach at the sensory processing level, since it helps the sensory development of children with ADHD, resulting in their language development, since it is interconnected with their ability to concentrate and with their understanding of social cues [24,29,79]. By applying techniques that include the use of movement and touch to improve language skills, the occupational therapist helps children to connect verbal and non-verbal communication through engaging them in motor experiences [92,93].

At the same time, occupational therapy has been shown to be particularly important for the development of executive functions such as self-regulation, working memory and attention, which are considered essential for children with ADHD to organise their linguistic expression, as they have difficulty maintaining a conversation, remembering instructions or organising their speech coherently. The occupational therapist helps to develop these skills by engaging children in structured activities, such as learning planning strategies and using virtual aids [24,29].

### 6.2. The Role of the Psychologist in Improving the Language Development of Children with ADHD

As children with ADHD struggle with language structure and organization, both their written and oral communication is affected [94]. To mitigate the difficulties, psychologists make use of cognitive behavioral therapy techniques and methods that help to enhance working memory and concentration [95].

At the same time, psychologists also help to improve pragmatic language, i.e., the ability to use language in social situations, conditions in which children with ADHD have significant deficits [94]. Strengthening emotional awareness as well as recognition of social cues can be systematically enhanced through psychological support for children with ADHD [15].

The role of the school and family context in enhancing the language development of children with ADHD is also considered important. The aforementioned goal is achieved through school psychologists, who work with the educational community to create individualized educational interventions. In addition, school psychologists provide advice to parents on how to support children’s language development at home [4]

Finally, psychoeducation helps children with ADHD to understand their difficulties and develop coping strategies on their own. Teaching children to use self-regulation techniques. such as slowing down their thinking before speaking and building confidence in their communication skills, they have been able to significantly improve verbal expression and comprehension [96,97,98]. By applying individualized intervention programs, psychologists practiced children in language self-regulation strategies, such as using inner speech and step-by-step formulation of their plans before oral expression [99]. At the same time, self-regulation interventions implemented within the educational environment help to improve concentration and working memory of children with ADHD, enhancing their comprehension and speech production skills [100].

Psychologists therefore play an essential role in developing the language skills of children with ADHD through social and educational interventions. By enhancing executive function, social communication and collaboration with other specialists, for example occupational therapists, psychologists help to improve children’s level of everyday communication as well as academic performance [81].

Across intervention studies, several methodological limitations should be noted. Many investigations rely on small sample sizes, lack randomization or active control groups, and provide limited information on long-term maintenance of treatment effects. As a result, although the reviewed interventions appear promising, conclusions regarding their effectiveness should be considered preliminary, underscoring the need for well-designed, controlled trials with longitudinal follow-up.

## 7. Discussion

The present literature review demonstrated the close relationship between ADHD and language development difficulties [101]. This relationship is multifaceted, arising from the core cognitive challenges (attention, working memory, executive functions) inherent in ADHD, which interfere with efficient language processing. The main domains of difficulty include phonological awareness, pragmatic understanding, executive functions, and written expression [24]. The difficulties are exacerbated by the deficits in attention, working memory and executive skills exhibited by children with ADHD [11].

The present literature review demonstrated the important role of occupational therapists and psychologists in mitigating the difficulties that children with ADHD present in language development. It also established the importance of implementing targeted, interventions aimed at improving these skills can bring about significant positive outcomes for children with ADHD [92]. Specifically, interventions that focus on enhancing phonological awareness, through phonemic awareness training, lead to improved reading skills [102]. At the same time, speech and language interventions contribute to oral and written expression, focusing specifically on improving the level of storytelling and speech structure of children with ADHD [103,104,105,106]. Interventions that enhance executive functions, such as working memory and speech organization, improve children’s ability to manage school demands, while the use of technological tools and social skills training enhance communication skills and social interactions of children with ADHD [103].

An important finding of the present review is the uneven distribution of evidence across linguistic domains. While phonology and pragmatics have been examined extensively, morphology and semantics remain comparatively underrepresented in high-quality empirical research. This imbalance does not necessarily reflect reduced clinical relevance, but rather highlights a significant gap in the current literature that warrants targeted investigation using fine-grained, domain-specific language measures. The above demonstrates that the implementation of integrated cognitive interventions is crucial in supporting children with ADHD [104]. These interventions, when individualised and combining educational, therapeutic and technological strategies, help to improve children’s language development, communication and academic performance [105,106,107]. A multidisciplinary approach and collaboration between teachers, parents and therapists is recommended to fully support people with ADHD and enhance their cognitive and language skills [79].

## 8. Conclusions

Children with ADHD often experience difficulties in language development, including phonological awareness, pragmatics, executive functions, and written expression [108,109,110,111]. Multidisciplinary collaboration [112,113,114,115] among teachers, parents, and therapists is essential to maximize the effectiveness of these interventions [116,117,118,119,120,121].

Several limitations constrain the interpretation of these findings. Heterogeneity in study designs, assessment tools, and participant characteristics limits generalizability. The predominance of cross-sectional studies prevents conclusions about developmental trajectories, while small sample sizes and potential publication bias may inflate effect sizes. Frequent comorbidity of ADHD with Developmental Language Disorder (DLD) or other learning disabilities complicates attribution of language deficits specifically to ADHD. Few studies have directly compared ADHD and DLD [113] groups using fine-grained morphosyntactic measures; for example, children with ADHD showed deficits on omnibus morphosyntax tasks but not on clinical probe tests of object clitic pronouns [17]. Finally, the relatively small number of eligible studies and the heterogeneity of study designs, assessment tools, and outcome measures limit the generalizability of the findings. In addition, the absence of a pre-registered protocol and the lack of standardized quality appraisal tools may have introduced bias, despite efforts to ensure transparency and methodological rigor. Finally, the predominance of studies conducted in Western, high-income contexts highlights the need for culturally and linguistically diverse research in this field. Despite these limitations, the present review provides a comprehensive synthesis of current evidence, highlighting key areas for intervention and future research, and offers valuable insights for supporting language development in children with ADHD.

## Data Availability

The data presented in this study are available on request from the corresponding author. The data are not publicly available due to privacy and ethical reasons.
